# Elucidation of the Effect of Solar Light on the Near-Infrared Excitation Raman Spectroscopy-Based Analysis of Fabric Dyes

**DOI:** 10.3390/molecules29215177

**Published:** 2024-10-31

**Authors:** Shannon Bober, Dmitry Kurouski

**Affiliations:** Department of Biochemistry and Biophysics, Texas A&M University, College Station, TX 77843, USA; shannonbober@tamu.edu

**Keywords:** NIeRS, fabric, colorants, fading, sun light

## Abstract

Colored textiles are valuable physical evidence often found at crime scenes. Analysis of the chemical structure of textiles could be used to establish a connection between fabric found at a crime scene and suspect cloths. High-performance liquid chromatography (HPLC) and mass spectroscopy coupled HPLC are traditionally used for the identification of dyes in fabric. However, these techniques are invasive and destructive. A growing body of evidence indicates that near-infrared excitation (λ = 830 nm) Raman spectroscopy (NIeRS) could be used to probe the chemical signature of such colorants. At the same time, it remains unclear whether environmental factors, such as solar light could lower the accuracy of NIeRS-based identification of dyes in textiles. In this study, we exposed cotton fabric colored with six different dyes to light and investigated the extent to which colorants fade during seven weeks using NIeRS. We found a decrease in the intensities of all vibrational bands in the acquired spectra as the time of the exposition of fabric to light increased. Nevertheless, utilization of partial least-squared discriminant analysis (PLS-DA) enabled identification of the colorants at all eight weeks. These results indicate that the effect of light exposure should be strongly considered by forensic experts upon the NIeRS-based analysis of colored fabric.

## 1. Introduction

Fabric, or other textiles, are often found during criminal investigations [1,2]. These pieces of evidence can be used in connecting and ruling out suspects. Forensic experts used several techniques, including microscopy and chromatography, to analyze such fabric [3,4]. Light microscopy can reveal morphological properties of fibers, as well as shed light on mechanistic damage of textiles that is often evident on such samples [5,6]. Morphological similarities in texture and damage pattern (thorning, cutting, and pulling) of fabric found at the crime scene and fabric possessed by the suspect can show a connection of the suspect to the crime scene or demonstrate absence of such connections. Chromatography, including high-performance liquid chromatography (HPLC) and mass spectroscopy-coupled HPLC (HPLC-MS), is capable of detecting illicit drugs and pharmacological substances present on fabric [3,7,8]. These techniques could be also used to determine the chemical structure of dyes if reference samples of such colorants are present in the forensic laboratory [3,9,10,11]. These results can straighten information about the fabric texture obtained from its morphological examination. Such dye-by-dye comparison is extremely laborious and expensive. Therefore, alternative techniques are often employed to analyze textile dyes.

Fluorescence spectroscopy and microscopy could be used to shed light on the chemical structure of the dyes present in fabric. Similar results can be provided by infrared (IR) spectroscopy [12,13]. However, both techniques are very sensitive to the nature of fiber materials because synthetic and natural fibers have drastically different fluorescence and IR spectra. The problem of both fabric and dye fluorescence can be overcome by surface-enhanced Raman spectroscopy (SERS) [3,9,10,11]. In this approach, noble metal nanostructures, such as gold colloids, are applied on the fabric [14,15,16]. Next, laser light is focused on the sample. Electromagnetic radiation generates coherent oscillations on the surface of nanostructures, also known as localized surface plasmon resonances (LSPRs), that enhance 106–108-fold Raman scattering from molecules present close to their surfaces [17,18,19,20,21]. As a result, SERS provides the single-level sensitivity in detection and identification of dyes [22]. Our group demonstrated that SERS could be used to detect and identify dyes present on hair [23,24]. Furthermore, SERS could be used to track the length of exposure of colored hair to soils, water submergence, and elevated temperatures [25,26]. In addition, SERS is broadly used in art conservation science to identify dyes and pigments in paintings [3,9].

Recently, our group demonstrated that utilization of near-infrared excitation in Raman spectroscopy (NIeRS) also allows for overcoming dye and fabric fluorescence [27]. This technique could be used to reveal both the chemical structure of fabric and identify chemical structure of dyes present in it [27]. Furthermore, NIeRS could be used to reveal body fluid and household contaminants present on such colored fabric [28]. However, it remains unclear whether environmental factors, such as solar light, could compromise the accuracy of NIeRS.

UV-based degradation or fading of dyes is a common problem not only in forensics, but also in art conservation sciences [3,10]. As dyes fade, their chemical structure and consequently color changes. As a result, visual perception of such artwork substantially deviates from its original appearance [3,10]. Therefore, a substantial effort in art conservation science is put to identify the structure and composition of pigments present in paintings and textiles [3,9,10,11]. Light-based fading of dyes possess a substantial pressure of SERS-based identification of dyes. For instance, Holman and Kurouski found that light exposure causes a progressive fading of hair colorants [29]. Nevertheless, utilization of partial least-squared discriminant analysis (PLS-DA) and corresponding spectroscopic libraries allows for overcoming the effect of light-driven colorants’ fading on hair [29].

Expanding upon this, we investigated the extent to which light exposure of fabric colored with six different dyes altered the accuracy of NIeRS-based identification of dyes. For this, colored fabric was exposed to sun in College Station, TX, USA for 7 weeks, Figures S1–S3. Every week, a fragment of the colored fabric is cut and analyzed using NIeRS. Finally, we used PLS-DA to investigate the accuracy of dye identification at each week of the fabric exposure to the solar light. This study models crime cases in which a piece of colored fabric was left exposed to light for more than two months period. We provide the answer as to whether analysis of colorants on such fabric could be feasible and beneficial to the crime scene investigation.

## 2. Results and Discussion

NIeRS spectra acquired from colored and intact cotton possess vibrational signatures of cotton and dyes (Figure S4 and Table 1). In our previous study, we demonstrated that spectroscopic fingerprints observed on colored fabric originate from dyes themselves [27].

**Figure 1:**
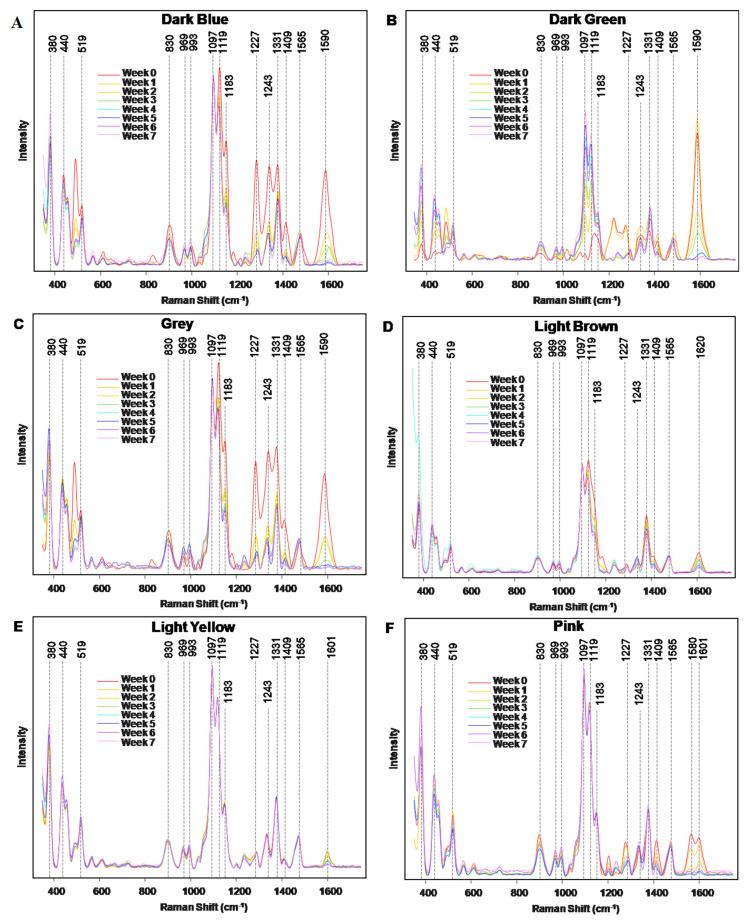
NIeRS spectra acquired from fabric colored with dark-blue, dark-green, grey, light-brown, light-yellow, and pink colorants before (week 0) and after 7 weeks of exposition to light. The excitation wavelength was 830 nm. Changes in the spectra are acquired from the fabric exposed to light and the intact fabric (week 0) point on the light-driven photodegradation of colorants on fabric.

In all cases, we observed a progressive decrease in the intensity of all vibrational bands as the duration of the light exposure increased (Figure 1 and Table 1). Furthermore, a major decrease in the intensity of vibrational bands that originate from the colorants was observed already at week 1, whereas by week 6–7 in most cases, these bands completely vanished (Figure 1). We also found shifts in the frequency of vibrational bands in some colorants, indicating chemical transformation in the dyes that were triggered by the light.

**Figure 2:**
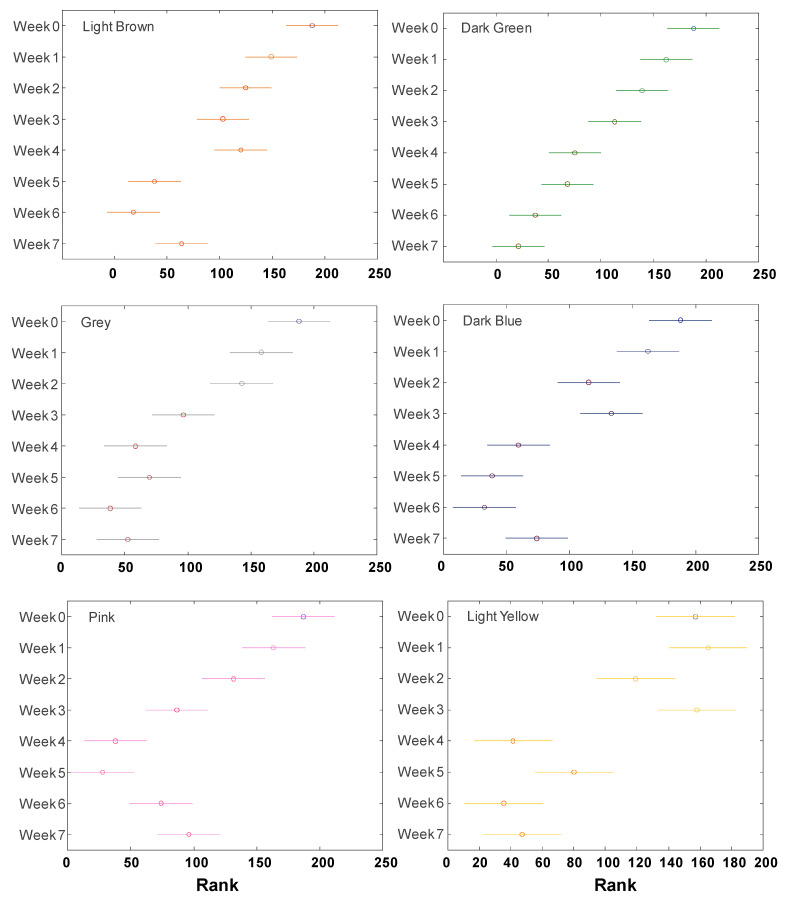
ANOVA of NIeRS spectra acquired from fabric colored with dark-blue, dark-green, grey, light-brown, light-yellow, and pink colorants before (week 0) and after 7 weeks of exposition to light.

We also found that changes in the intensity of vibrational bands that originate from the colorants could be used to identify the duration of the fabric exposure to the light, Figure 2. For this, we monitored changes in the intensity of 1610 cm^−1^ in the spectra that were normalized on a 1100 cm^−1^ band of cellulose. ANOVA of NIeRS spectra revealed a linear decrease in the intensity for green colorant, whereas more stepwise degradation was evident for light brown, grey, dark blue, and pink. ANOVA of light-yellow colorant indicated nearly consistent intensity between week 0 and week 3 that was followed by a rapid decrease at week 4–7. These results indicate that once the dye is identified, the duration of the fabric exposure could be determined using NIeRS considering similar weather conditions (Figures S2 and S3).

**Figure 3:**
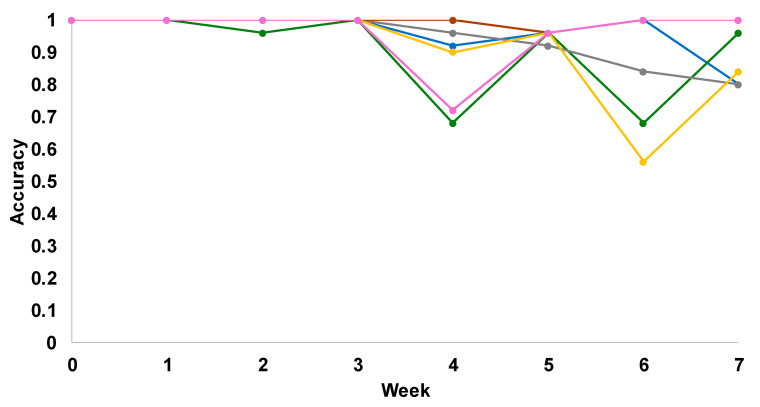
Prediction accuracy for NIeRS-based identification of grey, light-yellow, pink, dark-green, dark-blue, and brown colorants on cotton fabric.

Next, we utilized PLS-DA to investigate the accuracy of identification of each of the discussed above colorants. We found that up to week 3, all colorants could be correctly identified with 96–100% accuracy (Figure 3 and Figure S4 and Table 2). However, fading of colorants observed past week 4 drastically lowered the prediction accuracy of pink, light yellow, and grey colorants. It should be noted that the accuracy of identification of light-brown, grey, dark-blue, and light-yellow colorants did not change significantly at week 5, whereas the identification accuracy of pink and dark green substantially decreased (~70%). We also found that at week 6, the accuracy of identification of all colorants, except pink and dark blue, decreased to 60–80%. These results indicate that NIeRS could be used for highly accurate (96–100%) identification of all colorants on fabric exposed to light for less than 3 weeks, whereas less accurate identification of all colorants (~80%, on average) is feasible by week 7.

It is important to emphasize that Raman spectroscopy in general and NIeRS in particular provide information about the molecular fingerprints of analyzed samples [28]. In the case of uncolored fabric, we deal with the vibrational fingerprint of cellulose and lignin [30,31]. In the case of dyed fabric, two substances contribute to the acquired spectra: cellulose and colorants [27]. Numerous studies have shown that vibrational fingerprint of dyes directly depends on their structure [3,9,10,11,30,32]. Consequently, de novo calculations could be used to calculate Raman spectra of dyes if their structure is available. However, in most cases, such information will not be accessible to forensic experts. This limitation can be overcome by direct comparison of vibrational signature of a pure dye to the spectra of colored materials. This approach was used by Kurouski and Van Duyne to demonstrate that Raman spectroscopy directly probes Basic Blue 77 present in Sky Blue colorant developed by Ion [32]. However, considering thousands of such dyes commercially available on the market, spectroscopic references acquired in such studies become more accessible. Thus, one can expect that a spectroscopic library developed as a result of this work can be used by forensic experts across the country to identify dyes present on fabric.

## 3. Experimental

### 3.1. Materials

A 100% cotton shirt (scoured and bleached cotton) purchased from Fruit of the Loom was used as the blank canvas for all samples. “Dark blue”, “dark green”, “grey”, “light brown”, “light yellow”, and “pink” dyes (Kool Krafts, ASIN: B08N5HVXS8) were purchased from the Mosaiz Store (Vanstek Tie Dye Kit, 24 Colors Tie Dye Shirt DIY Fabric Dyes). These non-reactive colorants became highly popular around the world and are broadly used to dye fabric. Six different dyes were used to dye ~3-inch strips of fabric cut from the shirt: dark blue, dark green, gray, light brown, light yellow, and pink. Each strip of fabric dip was coated with enough dye to fully saturate the sample in a small sealable, plastic bag and left to sit for 1 h per manufacturer’s instructions. The fabric samples were rinsed under water until the water ran clear and left to dry for at least 24 h.

### 3.2. Methods for Exposure

Once samples were fully dried, ~1 cm of material was cut and placed in labeled, sealable plastic bags for later analysis as samples for week 0. Each of the six samples were stapled to a sturdy piece of cardboard, which was then secured to the outside of an unobstructed, south-west-facing brick wall using a VELCRO Brand Extreme Outdoor Medium Nylon Hook and Loop Fastener purchased from Legacy Ace Hardware (College Station, TX, USA). The minimum, maximum, and average temperatures were recorded along with any precipitation from the National Oceanic and Atmospheric Administration (NOAA). The maximum and average UV index was recorded along with the total daylight hours from Willy Weather, which sources their data from NOAA. All the weather data were collected daily and then calculated for each week. About ~1 cm of material from each of the samples was cut ~9 pm after sunset each Sunday and placed in a clean, sealed plastic bag. It should be noted that light is not the sole factor affecting the properties and spectral data of the samples; hair was also exposed to all environmental factors (humidity, insects, etc.) that can affect forensic sample at the crime scene.

### 3.3. Near-Infrared Excitation Raman Spectroscopy (NIeRS)

#### 3.3.1. Spectral Processing and Statistical Analysis

To collect the NIeRS spectra, a handheld Agilent Resolve Spectrometer was employed with a 830 nm laser that had a beam diameter of ~2 mm. Each spectrum was taken using a 1 s acquisition time and 495 mW laser power. Power density was 31.5 W/cm^2^. Spectra had an automatic baseline subtraction performed by the instrument. In total, 25 spectra were taken for each dye (dark blue, dark green, gray, light brown, light yellow, and pink) for each week. Scans were taken from different areas around the middle of each swatch for consistency between weeks.

#### 3.3.2. Spectral Processing and Statistical Analysis

The spectral processing was carried out in Matlab (MATLAB 7.5, Mathworks, Natick, MA, USA) equipped with PLS_Toolbox (Eigenvector Research, Inc., Manson, WA, USA). For the weekly dye comparisons, the spectra were area normalized and smoothed to the second polynomial (SavGol). PLS-DA models were trained with all spectra from the respective week and cross-validation was used to predict accuracy of the model to predict the dyes, as shown in Table 1. Classes were separated by the different colors used. The number of latent variables (LV) used for each week’s model was chosen by the lowest point on its respective RMSECV 1 graph.

For intensity comparisons of each dye, the spectra were all normalized at a ~1100 cm^−1^ peak, as that correlates to the cotton canvas used and smoothed to the second polynomial (SavGol) [7]. Kruskal–Wallis ANOVA was used to analyze the degradation of the dye over time. Spectra were normalized on this band because concentration of cellulose in fabric does not change as a result of light exposure. In this case, normalization on 1100 cm^−1^ allows for clear visualization of light-triggered changes in the intensities of vibrational bands that originated from dyes.

## 4. Conclusions

Our results demonstrate that NIeRS could be used to detect and identify dark-blue, dark-green, gray, light-brown, light-yellow, and pink colorants on fabric, as well as track fading rates of colored textiles. This information could be used to identify the duration of the exposure of such fabric to light. We also found that coupling PLS-DA enabled highly accurate identification of all colorants, as well as the duration of the fabric exposure to light during the first 3 weeks after the initiation of the light. Although the prediction accuracy slightly decreased for some of the tested colorants afterwards, PLS-DA was able to enable accurate identification of all colorants by week 7. These results indicate that NIeRS could be used to enhance forensic analysis of colored textiles exposed to solar light. It is important to expand such studies to examine the effect of shadowed environments as well as light at other geographic locations to establish the relationship between the dose of light and the magnitude of dye fading on fabric. It is also important to examine dye fading on other textiles. These experiments will be the subject of our future studies.

## Figures and Tables

**Table 1:** Vibrational bands in the NIRS spectra acquired from dyed and undyed cotton fabric.

Samples	Vibration Bands (cm^−1^)
Pink	379, 437, 494, 519, 567, 611, 648, 720, 828, 969, 995, 1095, 1119, 1183, 1200, 1215, 1331, 1353, 1377, 1409, 1469, 1579 *, 1601
Grey	380, 438, 454, 496, 520, 567, 611, 829, 969, 990, 1096, 1120, 1183, 1215, 1227, 1243, 1330, 1407, 1470, 1560, 1588
Light Yellow	380, 438, 455 *, 495, 519, 567, 610, 661 *, 724, 830, 969, 995, 1096, 1115, 1183, 1202 *, 1215, 1227, 1238, 1330, 1409, 1560, 1602
Dark Green	379, 437, 454*, 497, 518, 566, 606, 830, 966, 994, 1096, 1119, 1181, 1215, 1225, 1291, 1337, 1407, 1545, 1590
Dark Blue	379, 437, 453 *, 485, 517, 566, 602, 662 *, 723, 749, 815, 830, 969, 993, 1032 *, 1119, 1183, 1215, 1243, 1330, 1406, 1565, 1601
Light Brown	380, 438, 455 *, 492, 518, 565, 610, 701, 830, 969, 1000, 1033, 1100, 1119, 1183, 1190, 1215, 1235, 1243, 1331, 1407, 1565
Undyed	379, 437, 494, 518, 566, 610, 723, 900, 968, 995, 1095, 1118, 1149, 1237, 1289, 1336, 1378, 1475 and 1602

* = Evident in NIRS spectra of colored fabric but in the NIRS spectra of a blank cotton.

**Table 2:** True prediction rates (TPR) for each colored dye during weeks 0–7, along with the lowest TPR recorded for each week.

	Dark Blue	Dark Green	Gray	Light Brown	Light Yellow	Pink
Week	TPR (%)					
0	100	100	100	100	100	100
1	100	100	100	100	100	100
2	96	96	100	100	100	100
3	100	100	100	100	100	100
4	92	68	96	100	90	72
5	96	96	92	96	96	92
6	100	68	84	100	56	100
7	80	96	80	100	84	100

## Data Availability

The datasets used and/or analysed during the current study available from the corresponding author on reasonable request.

## Supplementary Materials

The following supporting information can be downloaded at: https://www.mdpi.com/article/10.3390/molecules29215177/s1, Figure S1. Colored fabric before (A) and after (B) 7 weeks of exposition to UV radiation. “Lt.Y”, light yellow, “Lt.Bio”, light brown, “P”, pink, “G”, gray, “DK.B”, dark blue, “DK.G”, dark green. Figure S2. Max and average values of UV radiation (top) and temperature (bottom) in College Station, TX. Figure S3. Average and total daylight hours during the 7 weeks of experiment. Figure S4. NIeRS spectra acquired from fabric colored with dark blue, dark green, grey, light brown, light yellow and pink colorants (week 0), as well as raw fabric (blank). Figure S5. LV plots of the models developed for identification of dyes on fabric exposed to solar light during weeks 1–7.
