# Supplementary material for: Elucidation of the Effect of Solar Light on the Near-Infrared Excitation Raman Spectroscopy-Based Analysis of Fabric Dyes
Source: Molecules. 2024 Oct 31;29(21):5177. doi: 10.3390/molecules29215177 (PMC11547680; doi:10.3390/molecules29215177)
Supplement: Supplementary file 1 [file molecules-29-05177-s001.zip › molecules-3256903-supplementary.pdf]

**Elucidation of the Effect of Solar Light on the Near-Infrared Excitation Raman Spectroscopy-Based Analysis of Fabric Dyes**

Shannon Bober[1] and Dmitry Kurouski[1*]

1. Department of Biochemistry and Biophysics, Texas A&M University, College Station, Texas 77843, United States

Supporting Information

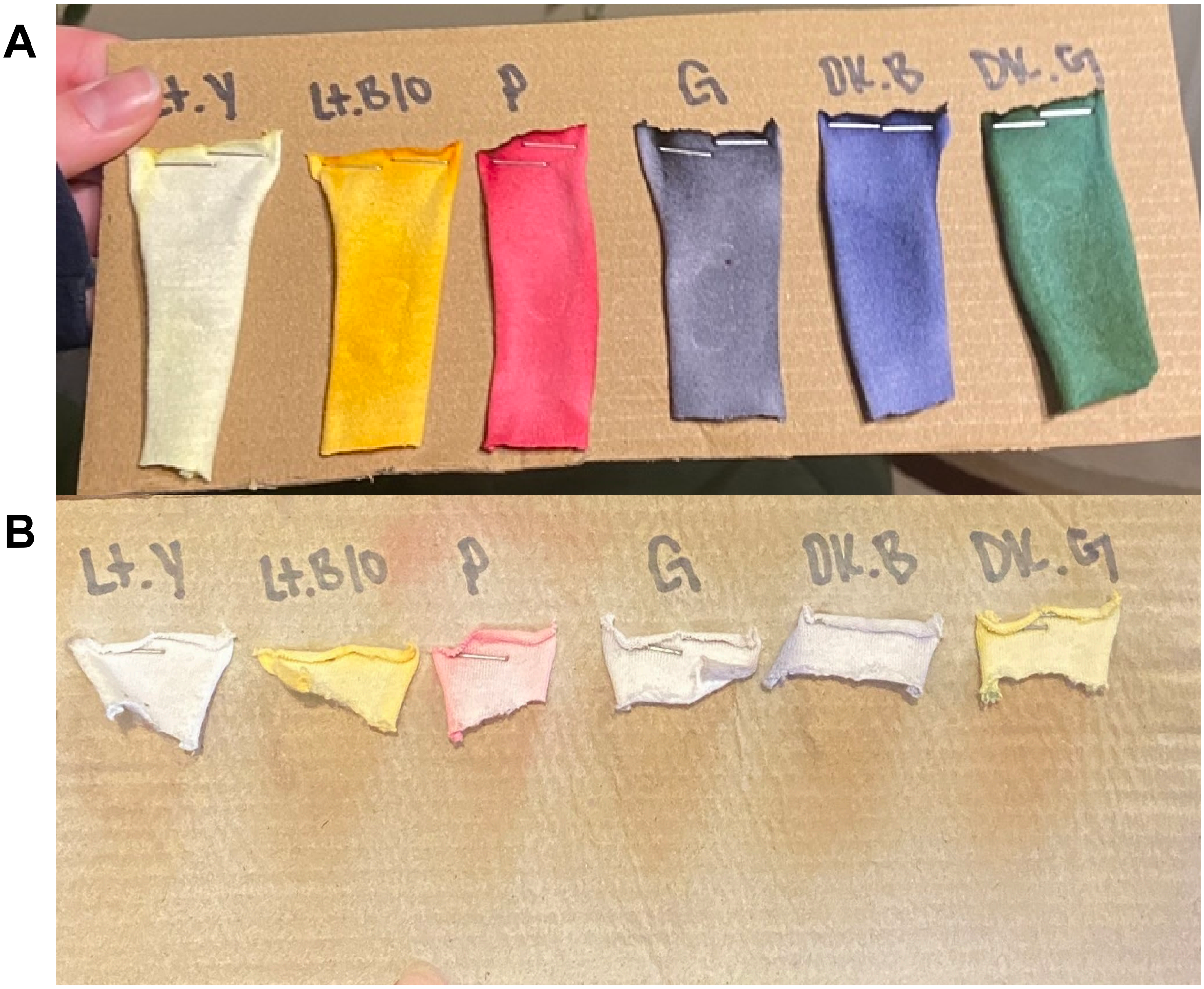


Figure S1. Colored fabric before (A) and after (B) 7 weeks of exposition to UV radiation. “Lt.Y”, light yellow, “Lt.Bio”, light brown, “P”, pink, “G”, gray, “DK.B”, dark blue, “DK.G”, dark green.

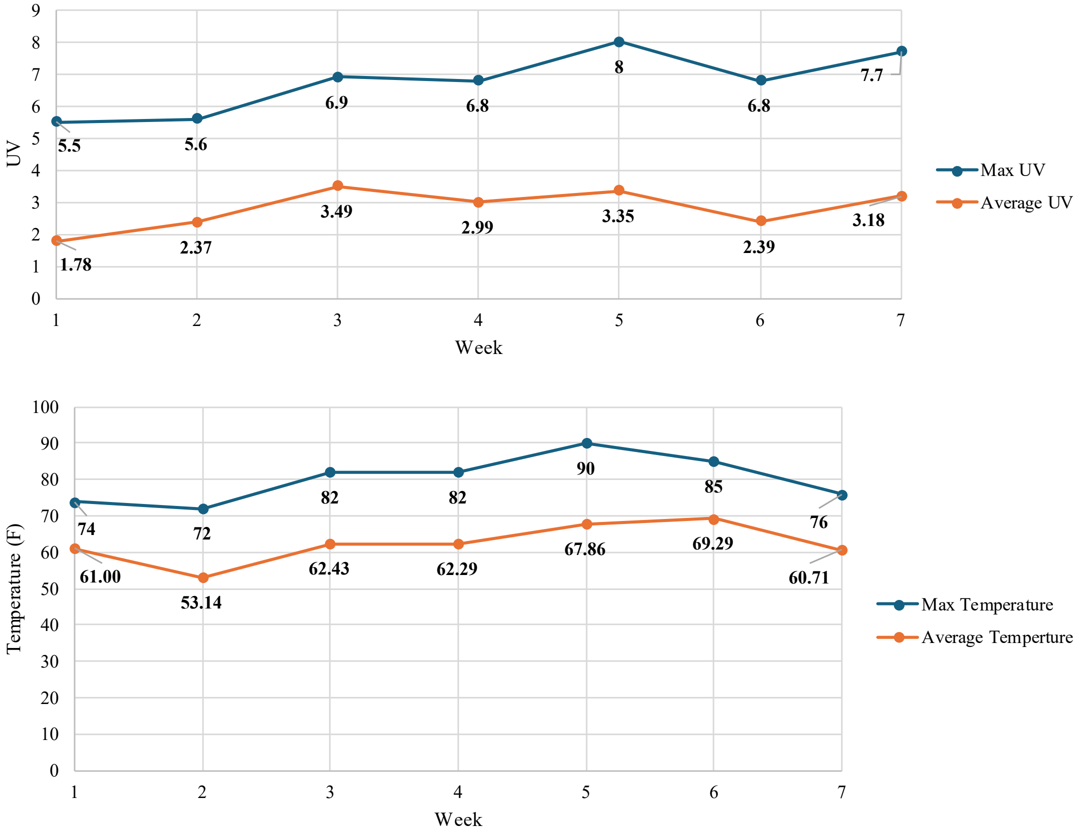


Figure S2. Max and average values of UV radiation (top) and temperature (bottom) in College Station, TX.

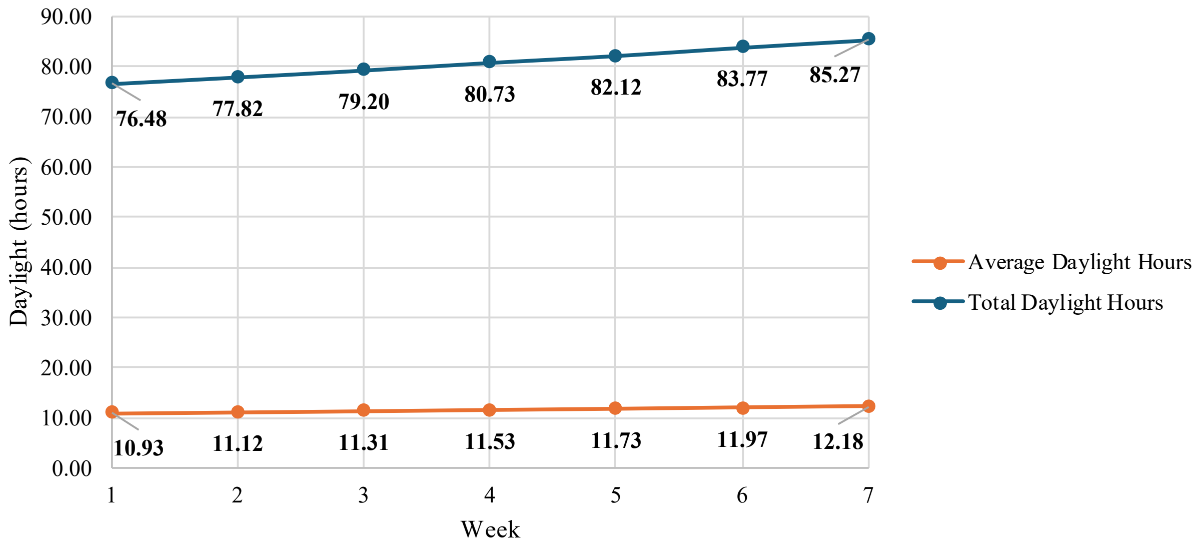


Figure S3. Average and total daylight hours during the 7 weeks of experiment.

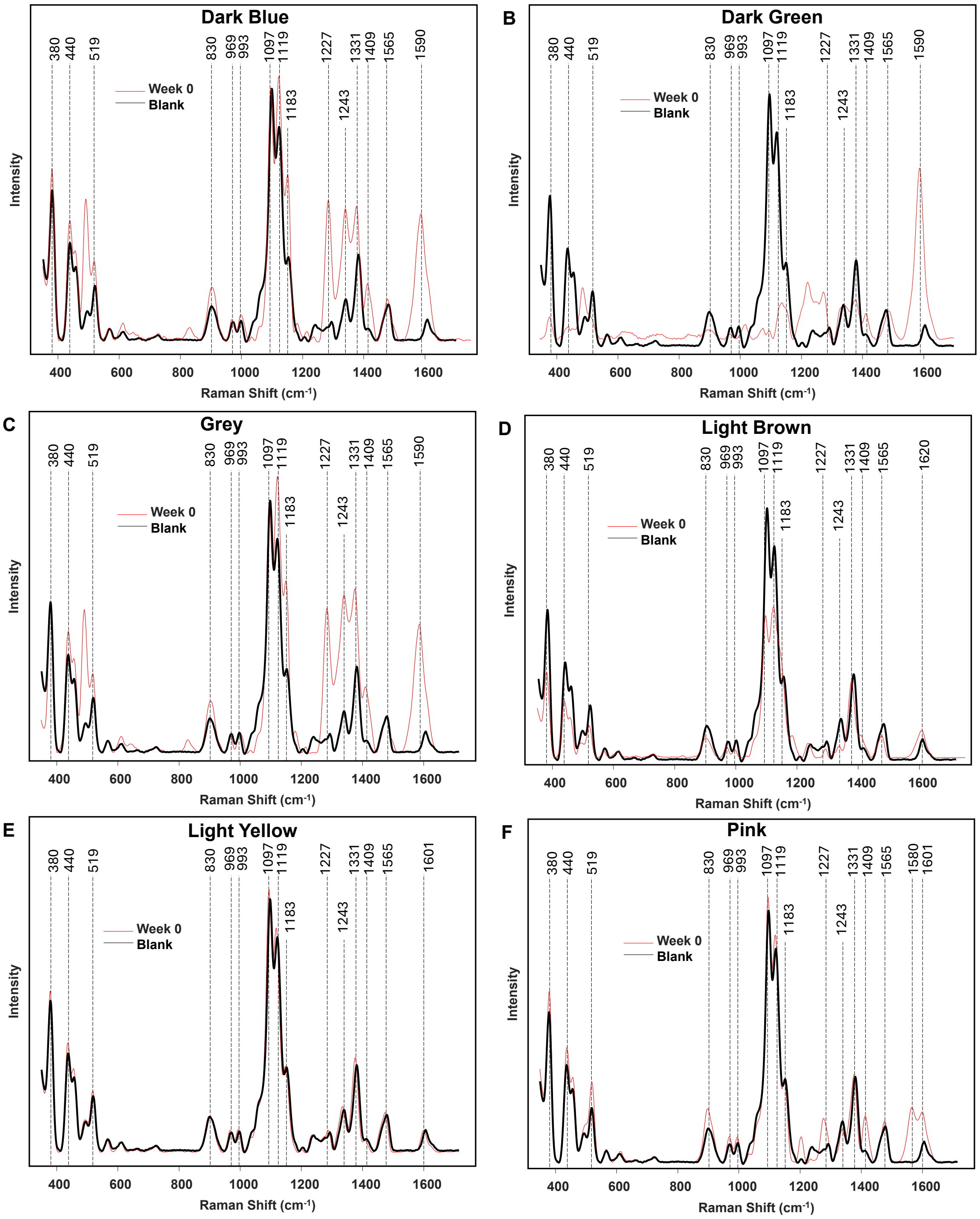


Figure S4. NIeRS spectra acquired from fabric colored with dark blue, dark green, grey, light brown, light yellow and pink colorants (week 0), as well as raw fabric (blank).

## Week 1

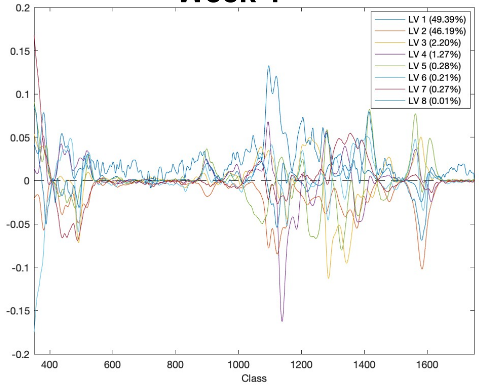


## Week 2

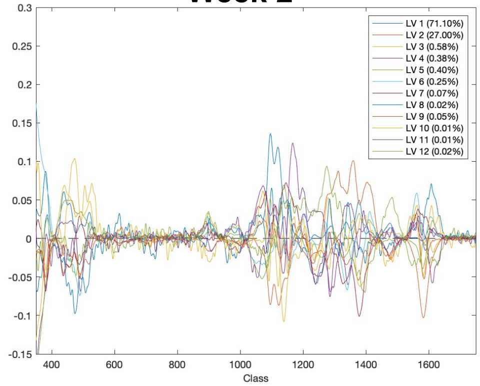


## Week 3

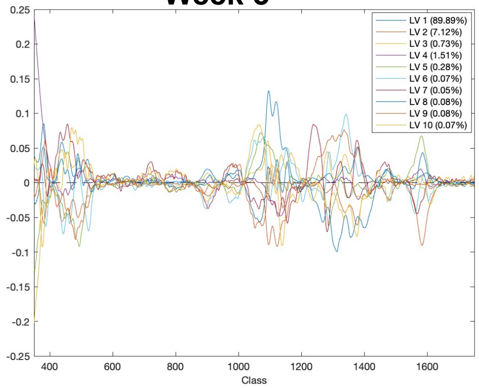


## Week 4

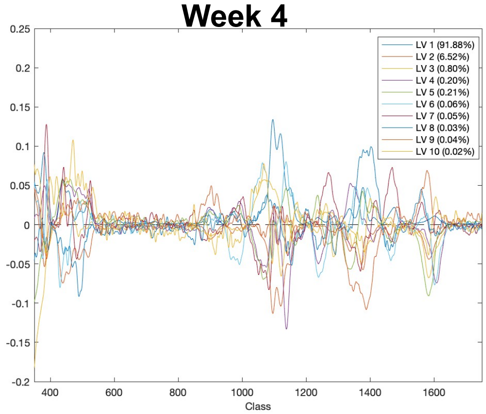


## Week 5

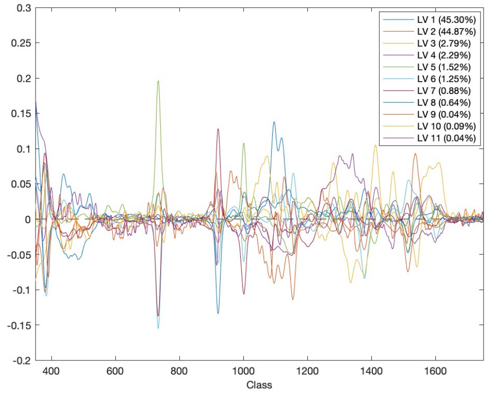


## Week 6

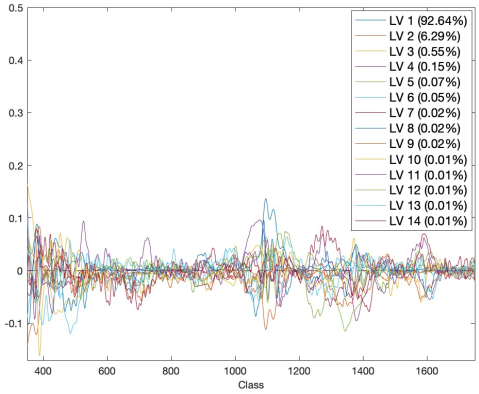


## Week 7

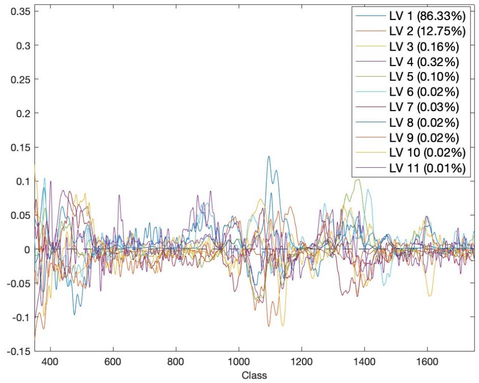

Figure S5. LV plots of the models developed for identification of dyes on fabric exposed to solar light during weeks 1-7.
